# A global-scale framework for quantifying the gut microbiomeʼs mediating role in environmental and personal determinants of health

**DOI:** 10.1016/j.eehl.2026.100236

**Published:** 2026-03-24

**Authors:** Yiwen Yuan, Jieying Ou, Xi Fu, Yuwei Tang, Yang Chen, Qi Deng, Yiqun Deng, Yu Sun

**Affiliations:** a State Key Laboratory of Swine and Poultry Breeding Industry, South China Agricultural University, Guangzhou 510642, China; b Guangdong Academy of Agricultural Sciences, Guangzhou 510640, China; c Guangdong Provincial Key Laboratory for the Development Biology and Environmental Adaptation of Agricultural Organisms, College of Life Sciences, South China Agricultural University, Guangzhou 510642, China; d Guangdong Provincial Engineering Research Center of Public Health Detection and Assessment, NMPA Key Laboratory for Technology Research and Evaluation of Pharmacovigilance, School of Public Health, Guangdong Pharmaceutical University, Guangzhou 510006, China; e Key Laboratory of Vegetation Restoration and Management of Degraded Ecosystems / Guangdong Provincial Key Laboratory of Applied Botany, South China Botanical Garden, Chinese Academy of Sciences, Guangzhou 510650, China; f South China National Botanical Garden, Guangzhou 510650, China

**Keywords:** Exposome, Mediation analysis, Environmental health, Persistent organic pollutants (POPs), Global health, Gut microbiome

## Abstract

The human gut microbiome mediates health risks from the exposome, but research focuses on diet and lifestyle, leaving the impact of environmental characteristics unclear. Furthermore, the microbiota’s quantitative, mediating role in linking the full exposome to disease is poorly understood. We conducted a global-scale association and mediation analysis of 13,463 American Gut Project participants, linking 128 environmental/personal factors to 10 gut microbial indices. We identified 390 significant but moderate associations (R^2^ < 0.03, FDR < 0.05) between exposome factors and the microbiota, revealing it is influenced by numerous small, cumulative effects. Our analysis confirmed expected patterns, such as reduced gut diversity with antibiotic use and exposure to pollutants like PM_2.5_ and SO_2_. However, it also revealed counterintuitive findings, notably that several hazardous exposures, including alcohol, airborne persistent organic pollutants (POPs), and mycotoxin deoxynivalenol, were associated with increased alpha diversity (FDR < 0.05). Our mediation analysis linking these factors to 24 self-reported health outcomes identified 1129 significant pathways (*p* < 0.05), confirming established links such as antibiotic-associated risk for irritable bowel syndrome (IBS) and the protective effects of vegetable consumption on allergies. Our analysis also revealed striking paradoxes: exposure to POPs increased inflammatory bowel disease (IBD) risk, partly via an increase in gut alpha diversity (1.5%–15.7% mediated effect), directly challenging the “higher diversity is better” paradigm. Our global-scale analysis provides the first comprehensive map of the gut microbiomeʼs mediating role in the human exposome, establishing a methodological blueprint for assessing the microbial contribution to the global burden of environmental disease.

## Highlights

• Global framework quantifies the gut microbiome's mediating role in health.
• Gut microbiome is shaped by numerous small, cumulative exposome effects.
• Hazardous exposures (POPs, alcohol, DON) paradoxically increase gut diversity.
• POPs increase IBD risk, partly mediated through this increased diversity.

## Graphical abstract

**Figure:**
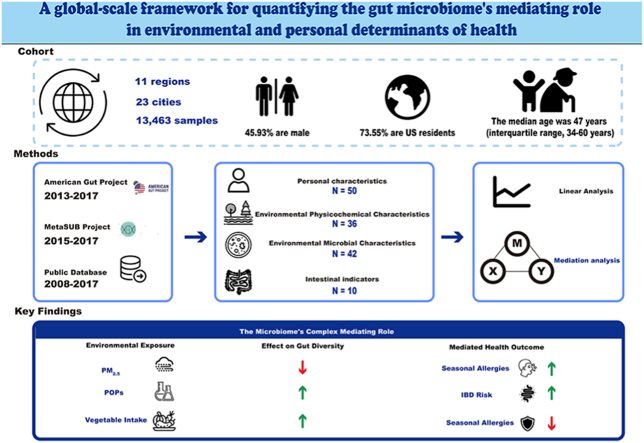

## 1 Introduction

The rising global burden of chronic non-communicable diseases is inextricably linked to a complex interplay of environmental exposures and personal characteristics [1]. The human gut microbiome represents a critical and dynamic interface in this relationship, metabolizing xenobiotics, modulating host immunity, and influencing physiological homeostasis [2,3]. Consequently, disruptions to this microbial ecosystem are increasingly associated with a wide array of pathologies, including inflammatory bowel diseases (IBD), metabolic disorders, and allergic diseases [[4], [5], [6]]. To properly address this, a systematic framework analogous to the Global Burden of Disease (GBD) study is urgently needed to dissect these complex, microbiota-mediated pathways at a global scale.

Current understanding is limited by several factors. First, while the impacts of diet and lifestyle on the gut microbiome are well-studied [[7], [8], [9], [10], [11], [12]], the effects of the broader exposome—encompassing environmental factors like persistent organic pollutants (POPs), mycotoxins, and the urban microbiome—remain comparatively underexplored. Although targeted studies have begun to shed light on the effects of specific pollutants, such as the link between prenatal tobacco exposure and alterations in the childhood gut microbiome [13], a comprehensive and systematic evaluation of the wider environmental landscape is still lacking. Second, many microbiome studies are conducted within a single city, region, or country, which significantly limits the generalizability of their findings. Because the gut microbiome is profoundly influenced by regional differences in diet, lifestyle, and environmental exposures, results from one population may not be applicable to others living in different global contexts [14,15]. Third, robust quantification of the gut microbiota’s mediating role is lacking for the broad range of environmental exposures. Specifically, quantitative estimates of the proportion of an exposure’s total health effect that is mediated through the microbiome are largely absent, limiting our understanding of its public health importance. Finally, the inherent complexity of microbiome data necessitates a multi-faceted analytical approach that moves beyond single metrics, a limitation of many previous association studies. To comprehensively capture this complexity, analyses should incorporate a diverse suite of ecological and functional indices, including alpha diversity (e.g., Shannon index, observed species), beta diversity (e.g., PCoA), broad structural features (e.g., the Firmicutes/Bacteroidetes ratio), and the abundance of key functional guilds (e.g., butyrate-producers, risk-associated taxa).

**Fig. 1:**
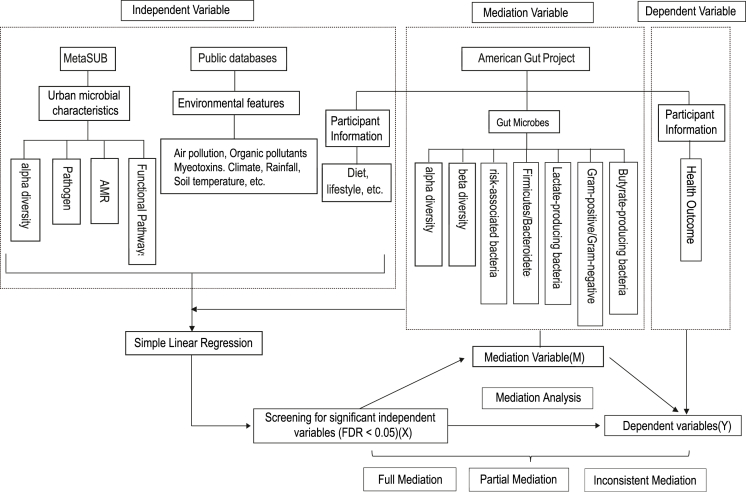
Technical approach for the global association and mediation analysis. The diagram outlines the study workflow. Independent variables were compiled from three main sources: the MetaSUB project (urban microbial characteristics), public databases (environmental features), and the AGP questionnaire (participant information). Mediation variables comprised 10 gut microbial indices derived from AGP fecal samples. Dependent variables were 24 self-reported health outcomes from the AGP questionnaire. The analytical pipeline involved initial univariate linear regressions to screen for significant associations (FDR < 0.05) between independent variables and both mediators and outcomes. Statistically relevant triplets of independent variables (X), mediators (M), and dependent variables (Y) were then subjected to formal mediation analysis to quantify direct (X→Y) and indirect (X→M→Y) effects, which were classified as full, partial, or inconsistent mediation. AGP, American Gut Project; FDR, false discovery rate.

To address these critical gaps, we conducted a global-scale association and mediation analysis based on data from 13,463 globally distributed participants within the American Gut Project, a large public cohort with substantial international participation [16]. We investigated the associations between 128 global environmental and personal characteristics, 10 gut microbiota indices, and 24 health outcomes, including gastrointestinal (GI), allergic, and metabolic disorders (Fig. 1). By systematically dissecting the direct effects of environmental/personal factors (i.e., the pathway of harm not involving the microbiome) versus their indirect effects (i.e., the pathway operating through changes in the gut microbiota), this study provides quantitative insights into these complex interactions. Our findings aim to advance the understanding of how diverse exposures shape the gut microbiome, thereby informing future preventive strategies, risk assessments, and potential microbiota-targeted therapeutic approaches.

## 2 Methods

### 2.1 Data acquisition and preprocessing from the American gut project

The American Gut Project (AGP) questionnaire data and participant fecal sample data (including genus-level OTU tables) were obtained from http://ftp.microbio.me/AmericanGut/latest/. The provided genus-level feature tables (representing Amplicon Sequence Variants [ASVs] aggregated to the genus level) were generated by the AGP/QIITA platform consistent with their standard processing protocols, employing QIIME 2 with DADA2 for sequence denoising and ASV generation, and taxonomic assignment against the Greengenes database (version 13_8). The initial data set comprised 24,694 samples; however, samples with missing latitude and longitude coordinates were first excluded, resulting in 18,024 samples. Subsequently, the questionnaire information was matched with the sample records, and only participant data that provided fecal samples were selected, leaving 14,217 samples. To handle missing data in the questionnaire, we applied a two-step filtering process. First, we conducted variable-wise filtering: any questionnaire variable with missing responses from more than 50% of the total participants was removed from the dataset. Next, we performed sample-wise filtering: any participant (i.e., sample) who had missing data for more than 50% of the remaining relevant variables was excluded. This rigorous filtering process resulted in a final cohort of 13,463 participants and 221 questionnaire variables for subsequent analysis.

To investigate associations between gut microbiota and geographic location, latitude and longitude coordinates from the original dataset were converted to city-level address information, facilitating regional characterization of gut flora. This was achieved using the Google Maps Geocoding API for reverse geocoding, with data processing performed in R (v4.4.2) via RStudio (v4.3.2). A Google API key was generated to access the Geocoding API. The R packages googleway and dplyr were utilized. Specifically, the geocode function (googleway) queried the API with participant coordinates. City-level address information was parsed from the returned JSON data and merged with the original dataset. To assign samples to a specific city, we used representative city-center coordinates as origins; all participant samples falling within a 5-km radius of a defined city center were considered to belong to that city.

### 2.2 Microbiome data collection and processing for the urban environment

Metagenomic data and associated metadata for global urban microbiomes were obtained from the MetaSUB project database [17] (Metagenomics and Metadesign of Subways and Urban Biomes, https://pangeabio.io/sample-groups/67e6b646-422e-4f61-9e9e-dac32caf23ba/analysis-results). This dataset included processed urban microbial taxonomic abundance tables, antimicrobial resistance (AMR) gene profiles, and functional pathway annotations. AMR gene abundances were quantified using reads per kilobase per million mapped reads (RPKM). The functional pathway data from MetaSUB were further screened and categorized using the MetaCyc database, yielding 41 pathway variables potentially relevant to human metabolism for our analysis. Given that the majority of MetaSUB samples originated from transport systems (n = 2674), with fewer from residences (n = 381), seaside locations (n = 178), schools (n = 142), hospitals (n = 45), and parks (n = 52), we focused exclusively on samples from transportation systems (primarily high-contact surfaces in subways and transportation hubs) to ensure consistency of environmental source.

To account for the uneven number of environmental samples per city in the MetaSUB dataset, we implemented a standardized subsampling procedure to create comparable, representative microbial profiles for each urban environment. This involved retaining 32 cities with sufficient sampling depth (>14 samples). The threshold of 55 samples per city was then selected for standardization because it represented the median sample size of these eligible cities. This specific cutoff allowed us to maximize the number of included locations while ensuring sufficient sampling depth for robust diversity estimation [18]. For cities with more than 55 samples, we applied an iterative random subsampling strategy to normalize the dataset to this target size. Crucially, this subsampling was repeated iteratively until the alpha diversity distribution of the selected 55-sample subset was not significantly different from that of the city’s full sample set (assessed using a two-sample Kolmogorov-Smirnov test, *p* > 0.05). This process ensured that the downsampled profiles remained ecologically representative of the full urban microbiome for each location. Alpha diversity metrics (Fisher’s alpha, Shannon entropy, and observed features) for these standardized city-level environmental metagenomes were then calculated using the vegan R package (v2.6.4) in R (v4.4.2) via RStudio (v4.3.2). The total abundance of obligate pathogens in the environment was calculated as the sum of *Brucella ovis*, *Brucella pinnipedialis*, *Salmonella enterica*, *Campylobacter jejuni*, *Listeria monocytogenes*, *Helicobacter pylori*, *Neisseria gonorrhoeae*, *Neisseria meningitidis*, *Streptococcus pyogenes*, and *Haemophilus influenzae*. The total abundance of facultative pathogens was calculated as the sum of *Acinetobacter baumannii*, *Klebsiella pneumoniae*, *Pseudomonas aeruginosa*, *Staphylococcus aureus*, *Clostridium perfringens*, *Enterococcus faecalis*, *Enterococcus faecium*, *Escherichia coli*, *Mycobacterium avium*, *Mycobacterium abscessus*, and *Streptococcus pneumoniae.* The obligate and facultative pathogens were mainly defined based on the NIAID-defined pathogens (https://www.niaid.nih.gov/research/niaid-biodefense-pathogens).

To link these urban environmental microbiome characteristics with human gut microbiota, we then mapped the geolocations of the 13,463 AGP participants to these MetaSUB cities. This integration resulted in a matched cohort of 2975 participants residing in 23 cities for which standardized MetaSUB environmental microbiome data were available. These samples were then subjected to the same questionnaire completeness filtering previously applied to the broader AGP cohort (i.e., exclusion if >50% of relevant questionnaire variables were missing), resulting in a final matched dataset of 1306 participants in 23 cities for the environmental microbiome association and mediation analyses.

### 2.3 Data sources and calculations of environmental characteristics

Environmental data was collected from multiple public databases (details in Tables S1 and S2). These data were obtained in various formats, including tabular, Esri grid (TIF/TIFF, NetCDF), and shapefile. Esri grid-formatted data, or shapefile data converted to Esri grid format, were processed using ArcMap (v10.8). For NetCDF files, relevant data layers were prepared before values corresponding to participant geolocations (city-level, as defined previously) were extracted. To address potential multicollinearity, a Spearman correlation analysis was conducted on all environmental and demographic features using the psych R package (v2.4.1) in R (v4.3.2). Variables exhibiting a Spearman’s rank correlation coefficient |ρ| > 0.85 with another variable were excluded.

To represent long-term environmental exposures relevant to each participant, we adopted a standardized temporal window. For time-varying environmental data, we collected information for the three calendar years immediately preceding the year of fecal sample collection for each AGP participant. For example, if a fecal sample was collected in 2015, environmental data from 2012, 2013, and 2014 were obtained, and the average annual exposure level over this three-year period was calculated for that participant. This approach was applied consistently unless otherwise specified.

In this study, exposure to six major mycotoxins—aflatoxin B1 (AFB1), fumonisin (FB), zearalenone (ZEN), deoxynivalenol (DON), ochratoxin A (OTA), and T-2 toxin—was estimated [19]. Source data comprised detection rates and concentrations of these mycotoxins in animal feeds and food commodities from various countries/regions spanning 2008–2017. As these raw data do not directly reflect each subject’s consumption, a two-step weighted estimation was employed. First, a regional representative concentration (*C*) for each mycotoxin (*k*) in each country/region (*j*) was calculated:

(1) $$C_{jk}=D_{jk}\times M_{jk}$$

Where, *D* is the detection rate, and *M* is the median concentration (μg/kg) of mycotoxin *k* in samples from country/region *j*.

Second, an estimated individual human exposure (*E*_*ik*_) for each participant (*i*) to each mycotoxin (*k*) was calculated based on their self-reported dietary intake frequencies for three primary food categories known to be sources of mycotoxin exposure. In the absence of individual biomarkers, we applied an empirical weighting scheme to approximate the relative contribution of each food group to total potential exposure. Weights were assigned to reflect the established hierarchy of contamination risk: grains, being the primary vector for major mycotoxins, were assigned the highest weight (W = 0.5), followed by dairy products (potential feed carry-over, W = 0.3) and nuts (W = 0.2). The formula used was:

(2) $$E_{k}=C_{jk}\times \sum_{f=1}^{3}\left(F_{if}\times W_{f}\right)$$

Where, *E*_*ik*_ corresponds to the estimated exposure (μg/day) for participant *i* to mycotoxin *k*; *C*_*jk*_ is the representative concentration (μg/kg) in the country/region *j* where participant *i* resides; *F*_*if*_ represents the reported intake frequency (times/day) for food category *f* by participant *i*, and *W*_*f*_ is the predefined dimensionless weight for that food category.

Annual data for air pollutants and airborne POPs were processed using the same three-year averaging method described above (i.e., averaging data from the three years preceding AGP sample collection). In instances where monitoring data were unavailable for a specific country/region within this precise three-year window, data from the most recent available preceding years were used as the best available estimate.

### 2.4 Calculation of personal characteristics for independent variables

Participant responses from the AGP questionnaire were numerically coded to facilitate quantitative analysis. Binary responses (e.g., “yes”/“true” or “no”/“false”) were coded as 1 and 0, respectively. For questions assessing food frequency or activity, responses were converted to a standardized scale approximating average daily occurrences: “never” = 0; “rarely (e.g., 2 times a month)” ≈ 0.066 (calculated as 2 occurrences/30.4 days per month); “occasionally (e.g., 1.5 times a week)” ≈ 0.214 (1.5 occurrences/7 days per week); “often (e.g., 4 times a week)” ≈ 0.571 (4 occurrences/7 days per week); and “every day” = 1. Other categorical or ordinal responses (e.g., Likert scales, multiple choice selections not related to frequency) were typically coded on a 0 to N−1 scale, where N is the number of options (e.g., a 5-option scale coded 0 to 4).

After initial coding, to mitigate multicollinearity and model instability in subsequent regression analyses, we performed a Spearman correlation analysis on all derived questionnaire variables. Variables with a Spearman’s rank correlation coefficient |ρ| > 0.85 with another variable were excluded. The remaining variables representing demographic and baseline characteristics (including age, gender, education level, and key self-reported dietary and physical health indicators) were designated as independent variables for subsequent regression models.

Finally, to organize the diverse array of predictors, all independent variables (including the 128 questionnaire-derived variables, environmental exposures, and environmental microbiome characteristics) were categorized into 13 overarching groups: personal characteristics, lifestyle habits, antibiotic/vaccination history, dietary frequency, alcohol consumption, vitamin/probiotic supplement usage, mycotoxin exposure, airborne POPs, air pollution metrics, other environmental characteristics, environmental microbial diversity/abundance, environmental AMR gene abundance, and environmental microbial functional pathway abundance.

### 2.5 Calculation of gut microbiota index

Alpha diversity metrics, including the Shannon Index, observed features, and Fisher’s alpha diversity, were calculated for each sample using QIIME2 (version 2024.2). Beta diversity was assessed by performing Principal Coordinates Analysis (PCoA) on a Bray-Curtis dissimilarity matrix generated from the OTU abundance table. The first two principal coordinates (PCoA1 and PCoA2) were used to represent major axes of community variation in subsequent analyses. Potential risk gut bacteria were identified based on taxa previously implicated in adverse health outcomes, drawing from two studies conducted in the Netherlands [14,20] and taxa associated with a low Gut Microbiota Health Index (GMHI) [21]. Firmicutes/Bacteroidetes (F/B) ratios were calculated directly from the OTU table. Gram-positive/Gram-negative (G+/G-) ratios were determined by assigning Gram status to taxa based on their known phylogeny and then summing their respective relative abundances. The total relative abundances of putative risk-associated bacteria, lactate-producing bacteria, and butyrate-producing bacteria were calculated by summing the relative abundances of constituent taxa from the OTU table. Risk-associated bacteria included *Eggerthella*, *Anaerotruncus*, and *Klebsiella*. Butyrate-producing bacteria included *Butyricimonas*, *Odoribacter*, *Anaerostipes*, *Anaerobutyricum*, *Agathobacter*, *Butyrivibrio*, *Coprococcus*, *Roseburia*, *Shuttleworthia*, *Butyricicoccus*, *Oscillibacter*, *Faecalibacterium*, *Flavonifractor*, *Pseudoflavonifractor*, *Subdoligranulum*, *Subdoligranulum variabile*, and *Eubacterium ventriosum*. Lactate-producing bacteria included *Bifidobacterium*, *Lactobacillus*, *Streptococcus*, *Enterococcus*, *Leuconostoc*, *Lactococcus*, and *Pediococcus*. These bacterial functional groups were defined based on previous publications. It is important to note that the risk-associated bacteria index is intended as an ecological marker of dysbiosis characterized by the expansion of opportunistic taxa, rather than evidence of confirmed active infection.

### 2.6 Calculation of personal health outcomes for dependent variables

In total, 36 variables corresponding to participants’ self-reported disease diagnoses or health conditions were identified as potential health outcomes from the AGP dataset. These included conditions such as body mass index (BMI), Small intestinal bacterial overgrowth (SIBO), IBD, and various allergies (Table S3). However, upon review, 12 of these variables were non-specific, representing broad categories rather than distinct disease states, or were otherwise unsuitable for direct disease-focused mediation analysis, such as autoimmune diseases, liver diseases, and kidney diseases. After excluding these 12 variables, the remaining 24 specific health conditions were retained as the final health outcomes for our mediation analyses.

### 2.7 Statistical analysis

#### 2.7.1 Group comparison statistics

All statistical comparisons of continuous microbial indices (e.g., alpha diversity, relative abundances of specific taxa) between two independent groups were performed using the non-parametric Mann-Whitney *U* test. To account for multiple hypothesis testing when numerous pairwise comparisons were made, the resulting *p*-values were adjusted to control the False Discovery Rate (FDR) using the Benjamini-Hochberg procedure. A result was considered statistically significant if the FDR-adjusted *p*-value was less than 0.05. Furthermore, to evaluate statistically significant differences in overall microbial community composition (beta diversity) across different categorical groups (e.g., geographic regions), Permutational Multivariate Analysis of Variance (PERMANOVA) was performed. This was conducted based on the Bray-Curtis dissimilarity matrix using the adonis2 function in the vegan R package, with 999 permutations.

#### 2.7.2 Linear regression analysis

To explore associations between individual environmental/personal characteristics (independent variables, IVs) and each gut microbial metric or health outcome (dependent variables, DVs), we performed a screen using bivariate linear regression. Each IV was regressed against each DV separately. These analyses were conducted using the regress command in Stata (version 18.0). To account for potential heteroskedasticity, heteroskedasticity-consistent standard errors (e.g., Huber-White) were estimated using the vce (robust) option. For each regression model, model fit was assessed using the adjusted R-squared (R^2^) value, and assumptions of linear regression were checked via residual analysis.

Statistical significance of the association between an IV and a DV was determined based on the 95% confidence interval (CI) for the regression coefficient (β) and FDR adjusted *p*-value. An association was considered statistically significant if the 95% CI for β did not include zero and the FDR-adjusted *p*-value was <0.05. Given the large number of bivariate regressions performed, controlling the FDR was crucial to minimize false positive findings arising from multiple comparisons. We therefore prioritized FDR-adjusted *p*-values for identifying genuinely significant associations in these exploratory univariate analyses. Only IVs meeting these significance criteria (FDR < 0.05) were considered for detailed reporting of univariate effects or as candidates for subsequent multivariable or mediation models.

#### 2.7.3 Mediation analysis

To investigate the mediating role of gut microbiota (M) in the relationship between environmental/personal characteristics (X) and health outcomes (Y), we conducted formal mediation analyses. Independent variables (X) selected for mediation were those showing a significant (FDR < 0.05) association with both a gut microbial metric (M) and a health outcome (Y) in prior univariate regressions. Gut microbial metrics (e.g., alpha diversity, PCoA axes, abundance of specific bacterial functional groups) served as potential mediators (M), and self-reported disease variables [e.g., irritable bowel syndrome (IBS), CDI, seasonal allergies] were the final dependent outcomes (Y). All continuous independent variables (X) were Z-score standardized prior to mediation analysis to enhance comparability. All mediation models were adjusted for a core set of confounders, including age and sex, to isolate the indirect effect from these demographic factors. Analyses were performed in R (v4.4.2) using RStudio (v4.3.2). The analytical approach differed based on the nature of the health outcome variable (Y):

Continuous Health Outcomes (Y): When the dependent variable was continuous, we followed the causal steps approach [22] to estimate the total effect (path c: X → Y), the effect of X on M (path a: X → M), and the effects of X (path c': X → Y|M) and M (path b: M → Y|X) on Y in a model controlling for both. The significance of the indirect effect (ab) was primarily assessed using bootstrapping (e.g., 5000 resamples) to generate 95% CIs. An indirect effect was considered significant if its 95% CI did not include zero. The proportion mediated was calculated as ab/c, where c is the total effect. Mediation was classified as partial if ab and c' had the same sign and both were significant, or inconsistent if ab and c' had opposite signs.

(3) $$Y=cX+e_{1}$$

(4) $$M=aX+e_{2}$$

(5) $$Y=c^{\prime }X+bM+e_{3}$$

Dichotomous Health Outcomes (Y): For dichotomous outcomes, we employed a mediation approach suitable for mixed regression models [23]. Path a (X → M) was estimated using linear regression. Paths involving the dichotomous outcome Y (X → Y, and X + M → Y) were estimated using logistic regression, yielding coefficients (c, c', b) on the log-odds scale. Given the different scales of coefficients from linear (for path a) and logistic (for path b) regressions, the indirect effect ab and its significance cannot be determined by simple multiplication and standard *t*-tests. We therefore utilized the Rmediation package [24] in R, specifically the medci function, to calculate product-of-coefficients-based asymmetric 95% CIs for the indirect effect (ab). An indirect effect was considered significant if this 95% CI did not include zero. The proportion mediated was reported as ab/c.

(6) $$Y^{\prime }=i_{5}+cX+\varepsilon_{5}$$

(7) $$M=i_{6}+aX+\varepsilon_{6}$$

(8) $$Y^{\prime \prime }=i_{7}+c^{\prime }X+bM+\varepsilon_{7}$$

(9) $$Y^{\prime }=\text{Logit}P\left(Y=1|X\right)=\ln\frac{P\left(Y=1|X\right)}{P\left(Y=0|X\right)}$$

(10) $$Y^{\prime \prime }=\text{Logit}P\left(Y=1|X\right)=\ln\frac{P\left(Y=1|M,X\right)}{P\left(Y=0|M,X\right)}$$

## 3 Results

### 3.1 Regional diversity and composition of the gut microbiome

We first analyzed 13,463 participants’ gut microbiome data from the American Gut Project (AGP). Detailed demographic baselines and long-term environmental exposure statistics for this cohort are provided in Text S1. To assess geographical variation in gut microbiota, we categorized samples into eleven regional groups, including the U.S. (further subdivided into West, South, Northeast, and Midwest), the UK, Australia, Canada, Switzerland, Belgium, Germany, and a collective group of “Other countries”. This group comprises all nations with fewer than 40 participants in the cohort, which were aggregated to avoid statistical instability associated with very small group sizes.

**Fig. 2:**
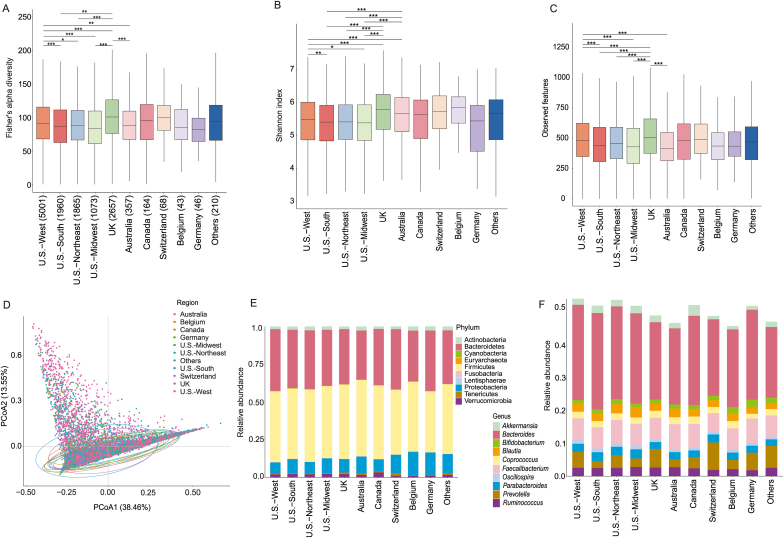
Geographic variation in gut microbiota composition and diversity across the AGP cohort. (A-C) Box plots comparing alpha diversity metrics across eleven geographic regions: (A) Fisher’s alpha diversity; (B) Shannon index; (C) Observed features. Box plot center lines represent medians, box bounds represent the interquartile range (IQR), and whiskers extend to 1.5 times the IQR. Asterisks indicate statistically significant differences between groups (Mann-Whitney *U* test; ∗*p* < 0.05, ∗∗*p* < 0.01, ∗∗∗*p* < 0.001). (D) Principal Coordinates Analysis (PCoA) plot based on Bray-Curtis dissimilarities, visualizing differences in overall community structure (beta diversity) between geographic regions. Each point represents an individual participant’s sample, colored by region. Ellipses represent 95% confidence intervals around the group centroids. The percentage of variance explained by each axis is shown in parentheses. Mean relative abundance of major microbial taxa at the (E) phylum level and (F) genus level, grouped by geographic region. For all panels, sample sizes for each region are indicated in the legend of panel A. The “Other countries” category represents a collection of nations with fewer than 40 samples each.

Alpha diversity varied significantly by geography (*p* < 0.001; FDR < 0.05), with participants in the UK exhibiting the highest microbial richness, surpassing those in the U.S. and Australia (Fig. 2A–C, Table S4). Significant regional differences were also apparent within the U.S., where participants from the Western region consistently showed higher alpha diversity across multiple metrics compared to those from the Southern region (*p* < 0.001).

PERMANOVA analysis confirmed a statistically significant, albeit modest, microbial compositional variation across regions (R^2^ = 0.12, *p* < 0.001; Fig. 2D–F). While geography explains a significant portion of the variance, the overlap of the ellipses in the PCoA plot underscores that a large amount of interpersonal variation remains, likely driven by the personal and lifestyle factors investigated in subsequent sections. The geographic variation was mainly driven by shifts in the relative abundance of dominant phyla like Firmicutes and Bacteroidetes, and key genera such as *Bacteroides* and *Prevotella* (Tables S5–7). The mean F/B ratio was significantly higher in participants from Australia (1.40) and the UK (1.36) compared to those from the U.S. (1.17) (*p* = 0.002, FDR = 0.03; Table S4). Furthermore, the abundance of putative butyrate- and lactate-producing bacteria was highest in participants from Belgium, significantly higher than observed in the U.S. and UK cohorts (*p* = 0.02; Table S4).

### 3.2 Environmental and personal characteristics affecting gut microbiota

**Fig. 3:**
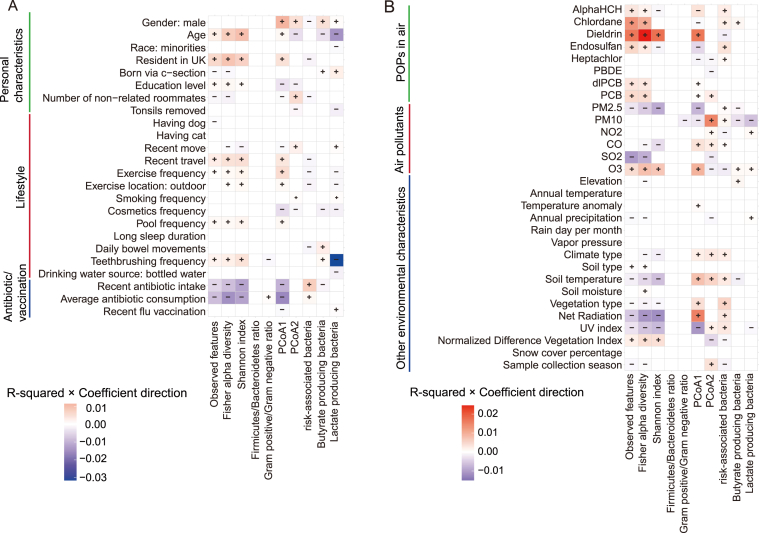
Associations between personal, lifestyle, and environmental factors and gut microbial indices. This heatmap displays the results of bivariate linear regression analyses linking a subset of personal and environmental characteristics (rows) with 10 gut microbial indices (columns). Rows are categorized by factor type: (A) personal characteristics, lifestyle habits, and antibiotic/vaccination history; and (B) airborne POPs, air pollutants, and other environmental characteristics. The color of each cell represents the proportion of variance explained (R^2^) multiplied by the direction of the association (coefficient sign): red indicates a positive association, while blue indicates a negative association. Color intensity is proportional to the R^2^. Only statistically significant associations (linear regression; FDR-adjusted *p* < 0.05) are colored. Plus (+) and minus (−) symbols are overlaid for enhanced clarity. The associations for dietary factors are shown in Fig. 4.

**Fig. 4:**
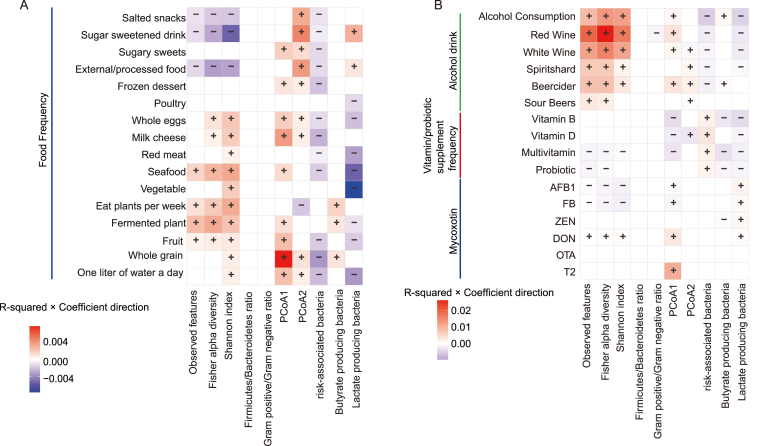
Associations between dietary and consumption factors and gut microbial indices. This heatmap, a continuation of Fig. 3, displays the results of bivariate linear regression analyses linking dietary characteristics (rows) with 10 gut microbial indices (columns). Rows are categorized by factor type: (A) food frequency and (B) alcohol, vitamin/probiotic, and mycotoxin exposures. The color of each cell represents the proportion of variance explained (R^2^) multiplied by the direction of the association (coefficient sign): red indicates a positive association, while blue indicates a negative association. Color intensity is proportional to the R^2^. Only statistically significant associations (linear regression; FDR-adjusted *p* < 0.05) are colored. Plus (+) and minus (−) symbols are overlaid for enhanced clarity.

**Fig. 5:**
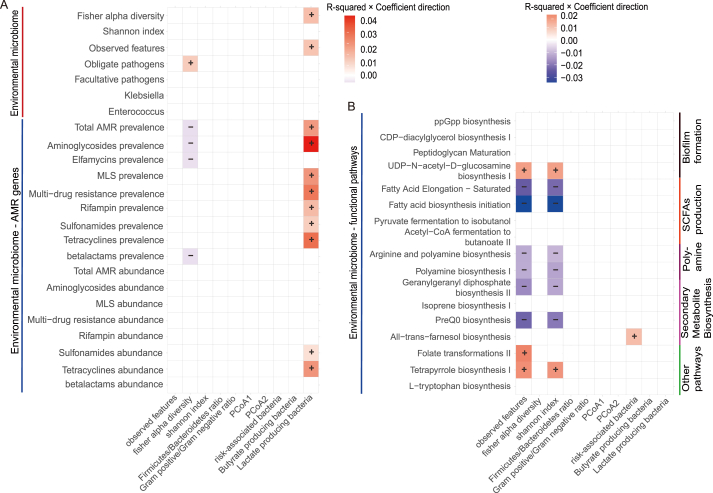
Associations of urban environmental microbiome characteristics with human gut microbial indices. The heatmaps show significant associations between characteristics of the urban environmental microbiome (rows) and gut microbial indices (columns) for the 1306 participants in the matched AGP-MetaSUB cohort. (A) Associations with environmental microbial diversity, specific taxa, and functional pathways. (B) Associations with environmental antimicrobial resistance (AMR) gene prevalence and abundance. The color of each cell represents the proportion of variance explained (R^2^) by the factor for that microbial index, multiplied by the direction of the association (coefficient sign). Red indicates a significant positive association, while blue indicates a significant negative association, with color intensity proportional to the R^2^. Only statistically significant associations (FDR-adjusted *p* < 0.05) are colored. Plus (+) and minus (−) symbols are overlaid for enhanced clarity of association direction.

To identify the most influential environmental and personal factors, we performed a bivariate screen using linear regression to model each of the 10 continuous gut microbiota indices as a function of the 128 characteristics. To focus on the most robust and meaningful signals, we defined a “significant association” as one that met a dual criterion of statistical significance (FDR < 0.05) and a minimum effect size (Adjusted R^2^ > 0.005). This rigorous filtering yielded 390 significant associations for further examination (FDR < 0.05; Fig. 3, Fig. 4, Fig. 5, Table S8).

Frequent alcohol consumption and airborne POPs levels emerged as the strongest positive predictors of gut alpha diversity (R^2^ = 0.0015–0.026; Fig. 4B, Table S8). This effect was consistent across various alcoholic beverages, including red wine, white wine, sour beers, beer cider, and total alcohol consumption, and various POPs (Fig. 3B), including Alpha HCH, Chlordane, Dieldrin, Endosulfan, dlPCB, and PCB. Other factors positively associated with increased alpha diversity included DON consumption (a mycotoxin; Fig. 4B), older age (Fig. 3A), residence in the UK (Fig. 3A), ozone levels (Fig. 3B), recent travel (Fig. 3A), and consumption of vegetables, fruits, and seafood (Fig. 4A)(R^2^ = 0.0005–0.024). Intriguingly, several traditionally considered hazardous exposures, such as alcohol consumption, POPs, and DON, were associated with increased gut alpha diversity.

To identify the specific taxa contributing to the increased species richness associated with POPs exposure, we compared the prevalence of each genus between participants in the high- and low-exposure quartiles for Chlordane and Dieldrin (Table S9). This analysis revealed a clear and consistent pattern: the increase in diversity was driven by the emergence of numerous taxa widely considered to be opportunistic pathogens or markers of a pro-inflammatory, dysbiotic gut environment. For Chlordane, the most pronounced increases in prevalence were seen for *Eggerthella* (+17.3%), a genus we pre-defined as risk-associated, and *Campylobacter* (+14.6%), a known potential pathogen. This pattern was reinforced by the significantly higher prevalence of other opportunistic genera, including *Megasphaera* (+14.6%), *Peptoniphilus* (+14.3%), *Anaerococcus* (+13.9%), *Finegoldia* (+12.6%), and the pro-inflammatory *Fusobacterium* (+5.9%). Notably, *Anaerotruncus* (+10.1%), another of our pre-defined risk-associated taxa, also became significantly more prevalent. A similar dysbiotic signature was observed with high Dieldrin exposure. This was associated with a striking increase in the prevalence of the pro-inflammatory, sulfite-reducing genus *Bilophila* (+8.8%). Other emerging taxa included *Oxalobacter* (+11.1%) and, once again, *Campylobacter* (+5.8%). These findings strongly suggest that the observed increase in alpha diversity is not a sign of a healthier ecosystem but rather reflects a dysbiotic shift characterized by the proliferation of potentially harmful bacteria.

Antibiotic intake was the primary factor reducing alpha diversity (R^2^ = 0.005–0.014; Fig. 3A). Other factors associated with decreased alpha diversity included net radiation and UV index (R^2^ = 0.0025–0.0115), as well as PM_2.5_ and SO_2_ concentrations (R^2^ = 0.0022–0.01; Fig. 3B). As expected, consumption of snacks, sugary beverages, and processed foods (R^2^ = 0.001–0.005; Fig. 4A) was also associated with lower diversity.

Gut microbial community structure (beta diversity) was significantly associated with a range of environmental and personal factors. The primary axis of variation, PCoA1, was most positively associated with exposure to the organic pollutant dieldrin (R^2^ = 0.016; Fig. 3B). Specifically, PCoA1 scores were positively associated with male gender, higher net radiation, ozone levels, and soil temperature, while being negatively associated with recent antibiotic use, higher UV index, and PM_2.5_ exposure (R^2^ = 0.007–0.0155; Fig. 3A and B). PCoA1 was positively correlated with the relative abundances of *Prevotella* and *Coprococcus*, and negatively correlated with *Bacteroides* and *Parabacteroides* (Pearson correlation, FDR < 0.05, |*r*| > 0.2; Table S7). Therefore, exposures such as dieldrin and ozone were associated with a microbial community composition enriched in *Prevotella* and *Coprococcus*, whereas factors like antibiotic use and PM_2.5_ exposure were associated with a state dominated by *Bacteroides* and *Parabacteroides*. The second axis of variation, PCoA2, was primarily associated with PM_10_ exposure (R^2^ = 0.0146). This axis was positively correlated with *Prevotella* and negatively with the beneficial genera *Faecalibacterium* and *Blautia*, suggesting that higher PM_10_ exposure may promote *Prevotella* at the expense of key butyrate-producing commensals.

The abundance of specific functional bacterial groups appears to be shaped by a multitude of factors, though individual characteristics typically explained only a small fraction of the observed variance (R^2^ < 0.01). For risk-associated gut microbiota, factors associated with increased abundance included antibiotic use, exposure to airborne POPs, and higher net radiation and ultraviolet index (R^2^ = 0.0021–0.0076; Fig. 3). Regarding butyrate-producing bacteria, positive associations were observed with male gender (Fig. 3A), higher daily bowel frequency, and a greater variety of plant intake per week (R^2^ = 0.0017–0.0046; Fig. 4A). Older age (R^2^ = 0.0035) and the intake of multivitamins and vitamin B supplements (R^2^ = 0.0023–0.003; Fig. 3A) were negatively associated with the abundance of butyrate-producing bacteria. Finally, factors negatively associated with lactate-producing bacteria included frequency of tooth brushing, male gender, consumption of vegetables, fruits, and seafood, PM_10_ exposure, and alcohol intake (R^2^ = 0.002–0.031; Fig. 3, Fig. 4), indicating diverse dietary, environmental, and lifestyle influences on this functional group.

We further assessed the impact of environmental microbiome characteristics on gut microbiota for the 1306 participants residing in 23 cities profiled by the MetaSUB project. Interestingly, higher environmental microbial richness, as measured by Fisher’s alpha diversity and observed features, was positively associated with the abundance of lactate-producing bacteria in the gut (R^2^ = 0.012–0.013; Fig. 5). Furthermore, a higher total abundance of obligate pathogens in the environment was positively correlated with gut microbiota Fisher’s alpha diversity (R^2^ = 0.01). Notably, a higher prevalence of antimicrobial resistance genes (ARGs) in the environment, including those conferring resistance to aminoglycosides, elfamycins, and beta-lactams, was negatively associated with both Fisher’s alpha diversity and observed features in the human gut (FDR < 0.05, R^2^ = 0.0048–0.0049). This finding highlights a potential public health concern in which the urban environmental resistome may directly impact human gut ecosystem stability. Further details on these and other associations with the urban microbiome, including specific environmental microbial functional pathways, are provided in Text S2.

### 3.3 Gut microbiota mediate effects of environmental and personal characteristics on health outcomes

**Fig. 6:**
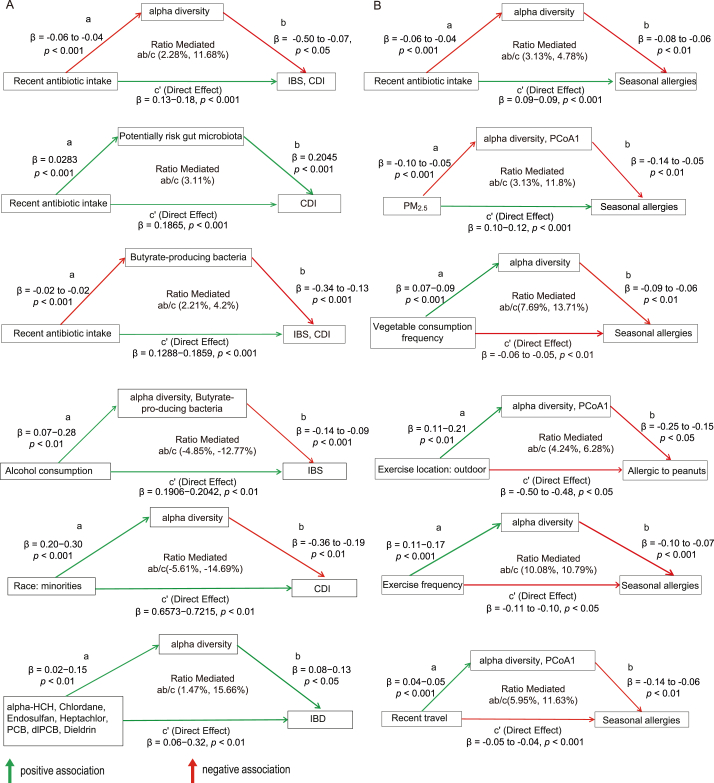
Gut microbiota mediate the effects of environmental and personal factors on health outcomes. The diagram illustrates a selection of significant mediation pathways linking environmental/personal characteristics (X, left boxes) to health outcomes (Y, right boxes), as mediated by gut microbial indices (M, top boxes). Arrows represent statistically significant paths, with the standardized path coefficient (β) and significance level (*p*-value) displayed for all direct and indirect path segments. Green arrows indicate a positive path coefficient (an increase in the predictor is associated with an increase in the outcome), while red arrows indicate a negative path coefficient. The “Ratio Mediated” refers to the proportion of the total effect accounted for by the indirect pathway (ab/c). Where applicable, ranges for β and the ratio mediated represent different models (e.g., using different microbial indices as mediators or different diseases as outcomes). (A) Representative mediation pathways for gastrointestinal (GI) diseases. (B) Representative mediation pathways for allergic diseases.

To elucidate the extent to which gut microbiota mediate observed associations between 128 environmental/personal factors and 24 health outcomes, we conducted formal mediation analyses (Fig. 6, Tables S10–11). Seasonal allergies (37.3%), lactose intolerance (16.0%), and IBS (14.2%) were among the most prevalent health outcomes in this cohort. Our analysis uncovered 1129 significant pathways linking these factors to health, including 738 partial and 391 inconsistent mediations. The following sections will focus on highly prevalent gastrointestinal and allergic diseases where gut microbiota is known to play significant roles, including IBS, CDI, IBD, and seasonal allergies.

#### 3.3.1 Gut microbial mediation effects for gastrointestinal diseases

As anticipated, recent antibiotic intake demonstrated strong direct adverse effects on both IBS and CDI (direct effect c' coefficients: IBS, 0.129–0.134; CDI, 0.172–0.186; Fig. 6A). These detrimental effects were consistently partially mediated by deleterious shifts in the gut microbiota, including reduced alpha diversity (indirect effect ab = 0.003–0.02), depleted butyrate-producing bacteria (0.003–0.008), and an increase in risk-associated bacteria (0.006). These indirect pathways collectively accounted for 2.28% to 11.68% (ab/c) of the total adverse effect of antibiotics on these GI conditions.

In contrast, alcohol consumption exhibited an inconsistent mediation pattern for IBS. While directly associated with an increased risk (c' = 0.19–0.20), its indirect pathway was beneficial, driven by the association of alcohol with increased gut alpha diversity and butyrate-producing bacteria. This resulted in a modest protective indirect effect (ab = −0.01 to −0.025) that offset approximately 4.85% to 12.77% (proportion mediated, ab/c) of the direct harm. Similarly, while minority race showed a direct association with increased CDI risk (c' = 0.66–0.72), it was concurrently associated with higher gut alpha diversity, which in turn was protective against CDI. This beneficial indirect pathway (ab = −0.036 to −0.106) mitigated 5.61% to 14.69% (ab/c) of the direct risk.

Exposure to a mixture of environmental POPs (alpha-HCH, Chlordane, Endosulfan, PCB, dlPCB, Dieldrin) was directly associated with an increased risk of IBD (c' = 0.06–0.32; Fig. 6A). Consistent with our general findings for POPs, exposure to these pollutants was associated with an increase in gut alpha diversity (a = 0.02–0.14). However, unlike the typically protective role of high diversity, this POPs-associated increase in alpha diversity was, in turn, positively associated with an increased risk of IBD (b = 0.07–0.12). This resulted in a detrimental indirect effect (ab = 0.003–0.012), contributing 1.47%–15.66% (ab/c) to the overall IBD risk. This suggests that the specific microbial species whose diversity is increased by these POPs may not be beneficial and could potentially contribute to, or serve as markers of, processes that exacerbate IBD risk, highlighting that not all increases in alpha diversity are necessarily health-promoting, especially in the context of pollutant exposure.

#### 3.3.2 Gut microbial mediation effects for allergic diseases

Consistent with their impact on GI health, recent antibiotic use also exacerbated seasonal allergy risk through direct effects and detrimental microbiota alterations (e.g., via reduced alpha diversity (ab/c = 3.13%–4.78%; Fig. 6B). Similarly, airborne PM_2.5_ directly increased seasonal allergy risk (c' = 0.104–0.116) and further contributed indirectly by reducing gut alpha diversity and shifting community structure (PCoA1) (ab = 0.004–0.012). These microbiota-mediated pathways accounted for 3.13% to 11.8% (ab/c) of the total PM_2.5_-associated allergy risk.

Conversely, health-promoting dietary and lifestyle factors were consistently protective against allergic diseases (Fig. 6B). Notably, higher vegetable consumption frequency showed a protective direct effect against seasonal allergies (c' = 0.054–0.06), and this benefit was partially mediated by an increase in gut alpha diversity (ab = −0.004 to −0.007, proportion mediated ab/c = 7.69%–13.71%). Similarly, lifestyle factors promoting environmental exposure—outdoor exercise, exercise frequency, and recent travel—were consistently protective against allergic diseases (Fig. 6B). These benefits were observed both directly (c' = −0.045 to −0.113 for outdoor exercise vs. seasonal allergies) and via beneficial gut microbiota modulation. Such lifestyles were associated with increased alpha diversity and health-associated shifts in community structure (PCoA1, e.g., increased *Prevotella*, decreased *Bacteroides*), leading to protective indirect effects (ab = 0.02–0.027, proportion mediated ab/c = 4.24%–6.28%). Collectively, these findings suggest that dietary choices rich in vegetables and active lifestyles that increase environmental microbial exposure may bolster resilience against allergies, in part by fostering a more robust, diverse, and well-structured gut microbiome.

## 4 Discussion

### 4.1 The “alpha diversity paradox” in environmental health

This study advances our understanding of microbiota-host-environment interactions by leveraging a large, global cohort to quantify the gut microbiome’s mediating role. We expanded the scope beyond traditional lifestyle factors to include a comprehensive set of environmental exposures and employed a diverse suite of microbial indices to provide a holistic assessment of the pathways linking the exposome to health.

A central finding of our analysis was the complex and often counterintuitive relationship between the exposome and gut microbial alpha diversity. While our results confirmed established patterns, such as reduced diversity with antibiotic use, the most striking discovery was the paradoxical association between increased alpha diversity and several traditionally hazardous exposures, including alcohol consumption, airborne POPs, and exposure to mycotoxin DON. While this challenges the simplistic “diversity-is-good” paradigm, our finding is not without precedent and contributes to a more nuanced ecological perspective.

For alcohol, while high-dose intake is clearly detrimental [25], several large cohort studies have noted a positive correlation between moderate alcohol consumption (particularly red wine) and alpha diversity, potentially linked to the effects of polyphenols on the microbiome [26,27], an effect consistent with observations in our cohort.

The positive associations with POPs and mycotoxins are less intuitive. However, this seemingly paradoxical finding is not without precedent, as several studies have reported that various hazardous materials are associated with increased gut alpha diversity, often interpreted as a sign of dysbiosis. Indeed, our analysis confirms this is a “harmful diversity”, driven by the emergence of opportunistic and pro-inflammatory taxa. We found that high exposure to POPs like Chlordane and Dieldrin was linked to a significantly higher prevalence of genera such as *Eggerthella*, *Campylobacter*, and *Bilophila*. This pattern is consistent with other studies; for instance, chronic exposure of mice to the organophosphate pesticide chlorpyrifos resulted in a significant increase in both species richness (Observed OTUs) and Shannon diversity [28]. Similarly, exposure to the herbicide atrazine also led to higher microbial richness in rodents [29]. An analogous pattern was observed with heavy metals, where exposure to cadmium in mice resulted in an increase in alpha diversity alongside shifts in potentially pathogenic bacteria like *Prevotella* and *Treponema* [30]. These consistent observations across different toxicants suggest a common underlying ecological mechanism. First, they may represent an adaptive microbial response where specific microbes capable of metabolizing these xenobiotic compounds proliferate [12]. Second, the toxicant could eliminate sensitive, dominant commensal species. This loss would create vacant ecological niches, allowing previously rare taxa to expand their populations. While either mechanism would numerically increase alpha diversity metrics, such a shift does not necessarily improve overall ecosystem resilience or host health [31]. It is crucial to emphasize that increased alpha diversity is not synonymous with a healthier gut state; the functional capacity could be more important [32]. These observations underscore the need to move beyond simple diversity metrics and consider functional profiles and specific microbial interactions when assessing the impact of environmental and lifestyle factors on gut health.

### 4.2 Mechanistic insights into exposure-driven dysbiosis

Beyond alpha diversity, our analysis showed that environmental exposures, particularly the legacy pollutant Dieldrin, remain significant drivers of gut beta diversity. We found that higher Dieldrin exposure was associated with a fundamental shift in microbial composition towards a *Prevotella*-enriched state. This is particularly noteworthy as a high *Prevotella*-to-*Bacteroides* (P/B) ratio is a well-established microbial signature linked to host inflammatory states [33], suggesting a plausible mechanistic pathway through which these persistent chemicals may continue to impact public health.

Our findings reveal that alcohol consumption was associated with higher gut alpha diversity and a greater abundance of butyrate-producing bacteria. These microbial features, in turn, were linked to a lower incidence of IBS. However, alcohol also exhibited a substantially stronger direct adverse effect, positively associating with elevated IBS risk. This seemingly paradoxical observation can be understood through a dose-dependent perspective, as highlighted by Caslin et al. [7]. Studies suggest that high alcohol intake is directly linked to the onset of inflammatory conditions, including IBS and allergies [34,35]. High-dose alcohol disrupts intestinal barrier integrity, exacerbating microbial dysbiosis and reducing diversity [36]. However, research on autoimmune diseases indicates that moderate alcohol consumption may reduce disease risk [37,38]. A potential explanation is that low-dose alcohol may modulate the gut microbiota to enrich beneficial taxa, promoting anti-inflammatory fatty acid production, including short-chain fatty acids (SCFAs) and polyunsaturated fatty acids (PUFAs) [39]. Thus, the impact of alcohol on gut microbiota—whether beneficial or detrimental—may be dose-dependent.

Our finding that exposure to POPs was associated with an increased risk of IBD, paradoxically mediated by an increase in gut alpha diversity, points to a state of detrimental dysbiosis rather than healthy diversification. This interpretation is supported by the specific compositional shifts in our data. Our differential abundance analysis revealed that high POPs exposure was associated with increases in several risk-associated and potentially pro-inflammatory taxa, like *Eggerthella*, *Anaerotruncus*, and *Klebsiella*. This pattern suggests a plausible ecological mechanism: POPs may act as selective pressures, creating an intestinal environment that is inhospitable to sensitive, dominant health-associated species [32]. The loss of these key players, which often occupy significant metabolic niches, could lead to a collapse in ecosystem stability. This creates an opportunity for rarer, more resilient, or opportunistic taxa—including those we identified as “risk-associated”—to expand and fill the vacant niches. While this process of replacement numerically increases the alpha diversity index, it simultaneously degrades the gut’s overall functional capacity.

To provide mechanistic examples from molecular studies, certain POPs like PCB-126 are known to activate the host’s aryl hydrocarbon receptor (AHR) pathway, which directly triggers intestinal inflammation and leads to the depletion of key beneficial commensals like *Bifidobacterium* and *Lactobacillus* [[40], [41], [42]]. These findings from in vitro and animal studies provide a concrete example of how POPs can create the selective pressures that underpin the dysbiotic, “diversified” state we observed in our cohort.

### 4.3 Interpreting modest effect sizes in complex microbiome ecosystems

A consistent observation throughout our analyses was that while many associations were statistically significant, the proportion of variance explained (R^2^) by any single exposome factor was modest, typically below 3%. This finding, while common in large-scale microbiome and epidemiological research, warrants careful interpretation. It is crucial to recognize that the gut microbiome is a complex ecosystem influenced by a multitude of factors, not a system dominated by a few strong drivers. Therefore, our results are consistent with a model where the microbiome’s structure is shaped by the cumulative burden of dozens of small, additive effects from diet, lifestyle, and environmental exposures, an architecture similar to that observed for other complex biological traits [43]. Furthermore, from a public health perspective, even a small effect size can be highly significant. When an exposure is ubiquitous, a factor explaining even 1% of the variance can translate to a substantial population-attributable risk, a principle central to preventive medicine [44,45]. Thus, our findings should be interpreted not as identifying singular “smoking gun” exposures, but as mapping a complex web of numerous, small but potentially impactful influences on the gut microbial ecosystem.

Another consistent observation across our mediation analyses was that the proportion of the total health effect mediated by the gut microbiota was often modest (typically <15%), despite being statistically significant. This finding is likely attributable to both biological and methodological factors. Biologically, the gut microbiota is just one component in a complex network of host-environment interactions, and our broad microbial indices may not capture the effects of specific keystone species or critical metabolites [46,47]. Furthermore, standard linear mediation models may not fully account for complex feedback loops, and inherent measurement error in both exposome and microbiome data likely attenuates the observed strength of these indirect pathways [34]. Therefore, our results should be interpreted as highlighting the gut microbiota as a crucial, but not exclusive, mechanistic link, underscoring the need for future studies using functional metagenomics and longitudinal designs.

### 4.4 Study limitations and future directions

Certain limitations should be acknowledged in this study. First, it is essential to underscore that our findings are based on a cross-sectional design, and therefore, the mediation pathways identified represent statistical associations rather than proven causal relationships. Formal mediation analysis relies on key assumptions, most critically the absence of unmeasured confounding between the exposure-mediator, mediator-outcome, and exposure-outcome relationships. While we adjusted for several key demographic factors, the potential for residual confounding from unmeasured variables (e.g., detailed medication use, genetic predispositions, specific dietary patterns not captured by food-frequency questions) remains. Consequently, our results should be interpreted as robust hypotheses about the mediating role of the microbiome that require validation in longitudinal and experimental studies to establish causality. Second, our analysis focuses on a unidirectional pathway (Exposure → Microbiome → Disease) and does not explore potential bidirectional feedback loops, such as the influence of a disease state on the gut microbiota, which would be best addressed with longitudinal data. Third, our study’s reliance on self-reported health outcomes from the AGP questionnaire is a significant limitation, introducing potential for recall bias and misclassification. Such errors are often non-differential, which would likely attenuate our findings and cause an underestimation of the true mediation effects. However, the possibility of differential bias (e.g., if reporting is linked to an exposure) cannot be excluded. Therefore, our results should be viewed as strong, hypothesis-generating findings that require confirmation in studies using clinically confirmed diagnoses. Fourth, the use of 16S rRNA gene sequencing, while enabling broad taxonomic profiling, restricts direct assessment of microbial function. While genus-level shifts provide robust ecological signals, this method lacks species- or strain-level resolution. Therefore, associations with risk-associated bacteria should be interpreted as ecological markers of dysbiosis rather than definitive evidence of specific pathogen proliferation. Fifth, despite its global participation, the AGP cohort is skewed towards individuals from developed nations, and the integration with MetaSUB data was limited to a smaller subset of participants. Consequently, while our large-scale computational analysis provides valuable insights, our findings represent robust hypotheses that warrant further validation in diverse, clinically-adjudicated cohorts, ideally with longitudinal designs and shotgun metagenomics.

In conclusion, by systematically quantifying the gut microbiome’s mediating role, this study provides a new, comprehensive map of the pathways linking the human exposome to health. Our findings reinforce the microbiome’s central importance in environmental health and provide a robust framework and a rich set of testable hypotheses to guide future research and the development of microbiota-targeted interventions.

## CRediT authorship contribution statement

**Yiwen Yuan:** Writing – original draft, Visualization, Validation, Software, Methodology, Formal analysis, Data curation, Conceptualization. **Jieying Ou:** Writing – original draft, Visualization, Validation, Formal analysis, Conceptualization. **Xi Fu:** Writing – review & editing, Validation, Supervision. **Yuwei Tang:** Writing – original draft, Visualization, Software, Investigation, Conceptualization. **Yang Chen:** Software, Resources, Methodology, Investigation, Formal analysis, Conceptualization. **Qi Deng:** Writing – review & editing, Supervision, Resources, Data curation. **Yiqun Deng:** Writing – review & editing, Validation, Supervision. **Yu Sun:** Writing – review & editing, Validation, Supervision, Funding acquisition.

## Declaration of competing interest

The authors declare that they have no known competing financial interests or personal relationships that could have appeared to influence the work reported in this paper.
