# Supplementary material for: A global-scale framework for quantifying the gut microbiomeʼs mediating role in environmental and personal determinants of health
Source: Eco Environ Health. 2026 Mar 24;5(2):100236. doi: 10.1016/j.eehl.2026.100236 (PMC13087684; doi:10.1016/j.eehl.2026.100236)
Supplement: Multimedia component 1 [file mmc1.docx]

Supplementary Information for

**A global-scale framework for quantifying the gut microbiomeʼs mediating role in environmental and personal determinants of health**

Yiwen Yuan^a,b,c,1^, Jieying Ou^a,c,1^, Xi Fu^d^, Yuwei Tang^a,c^, Yang Chen^a,c^, Qi Deng^e,f^, Yiqun Deng^a,b,^*, Yu Sun^a,c,^*

^a^ State Key Laboratory of Swine and Poultry Breeding Industry, South China Agricultural University, Guangzhou 510642, China.

^b^ Guangdong Academy of Agricultural Sciences, Guangzhou 510640, China.

^c^ Guangdong provincial key laboratory for the development biology and environmental adaptation of agricultural organisms, College of Life Sciences, South China Agricultural University, Guangzhou 510642, China.

^d^ Guangdong Provincial Engineering Research Center of Public Health Detection and Assessment, NMPA Key Laboratory for Technology Research and Evaluation of Pharmacovigilance, School of Public Health, Guangdong Pharmaceutical University, Guangzhou 510006, China.

^e^ Key Laboratory of Vegetation Restoration and Management of Degraded Ecosystems / Guangdong Provincial Key Laboratory of Applied Botany, South China Botanical Garden, Chinese Academy of Sciences, Guangzhou 510650, China.

^f^ South China National Botanical Garden, Guangzhou 510650, China.

^1^These authors contributed equally to this work.

*Corresponding authors.

Email: [sunyu@scau.edu.cn](mailto:sunyu@scau.edu.cn) (Y. Sun), [yqdeng@scau.edu.cn](mailto:yqdeng@scau.edu.cn) (Y. Deng).

Results

**Text S1. Baseline characteristics and environmental exposures of the study cohort**
The subjects included in this study had a balanced gender distribution (male, 45.93%). The majority of participants were middle-aged or older, with a median age of 47 years (IQR, 34–60 years). More than half had not taken antibiotics (66.27%) or taken a flu vaccination (55.9%) in the past year. We found that 74.79% of the participants drank alcohol, including red wine (38.39%), white wine (28.07%), spirits/hard liquor (24.87%), beer/cider (30.46%), and sour beers (4.16%). Regarding long-term environmental exposures, the 3-year average annual temperature for participants was 12.88°C, with a 3-year average monthly rainfall of 67.83 mm. The median 3-year average PM₂.₅ exposure preceding sample collection was 2.27 × 10⁻⁵ kg/m² (IQR: 1.97 × 10⁻⁵ to 2.50 × 10⁻⁵ kg/m²). For PM₁₀, the median exposure was 17.308 μg/m³ (IQR: 14.028 to 27.575 μg/m³), and the median NO₂ exposure was 5.88 × 10¹⁵ molecules/cm² (IQR: 4.96 × 10¹⁵ to 7.47 × 10¹⁵ molecules/cm²).

**Text S2. Extended associations between environmental microbial functional pathways and gut microbiota**
Beyond the environmental resistome, functional pathways of the urban microbiome also exhibited correlations with human gut microbiota. Higher abundance of environmental microbial functional pathways related to fatty acid production (FDR < 0.05, R² = 0.024–0.034), polyamine synthesis (FDR < 0.05, R² = 0.01–0.012), and queuosine production (FDR < 0.05, R² = 0.012–0.023) was negatively associated with both Fisher's alpha diversity and observed features in the human gut. Conversely, environmental microbial pathways for tetrapyrrole biosynthesis I and UDP-N-acetyl-D-glucosamine biosynthesis I were positively associated with human gut Fisher's alpha diversity and observed features (FDR < 0.05, R² = 0.015–0.018). While these findings highlight potential, underexplored links between the functional capacity of the urban microbiome and human gut microbial diversity, they should be interpreted with caution given the modest proportion of variance explained by these environmental features.
